# Risk factors associated with stress, anxiety, and depression among university undergraduate students

**DOI:** 10.3934/publichealth.2021004

**Published:** 2020-12-25

**Authors:** Mohammad Mofatteh

**Affiliations:** 1 Lincoln College, University of Oxford, Turl Street, Oxford OX1 3DR, United Kingdom; 2 Merton College, University of Oxford, Merton Street, Oxford OX1 4DJ, United Kingdom; 3 Sir William Dunn School of Pathology, University of Oxford, South Parks Road, Oxford OX1 3RE, United Kingdom

**Keywords:** mental health, public health, stress, anxiety, depression, students, university, undergraduate

## Abstract

It is well-known that prevalence of stress, anxiety, and depression is high among university undergraduate students in developed and developing countries. Students entering university are from different socioeconomic background, which can bring a variety of mental health risk factors. The aim of this review was to investigate present literatures to identify risk factors associated with stress, anxiety, and depression among university undergraduate students in developed and developing countries. I identified and critically evaluated forty-one articles about risk factors associated with mental health of undergraduate university students in developed and developing countries from 2000 to 2020 according to the inclusion criteria. Selected papers were analyzed for risk factor themes. Six different themes of risk factors were identified: psychological, academic, biological, lifestyle, social and financial. Different risk factor groups can have different degree of impact on students' stress, anxiety, and depression. Each theme of risk factor was further divided into multiple subthemes. Risk factors associated with stress, depression and anxiety among university students should be identified early in university to provide them with additional mental health support and prevent exacerbation of risk factors.

## 1. Introduction

Mental health is one of the most significant determinants of life quality and satisfaction. Poor mental health is a complex and common psychological problem among university undergraduate students in developed and developing countries [1]. Different psychological and psychiatric studies conducted in multiple developed and developing countries across the past decades have shown that prevalence of stress, anxiety, and depression (SAD) is higher among university students compared with the general population [2]–[4]. It is well established that as a multi-factorial problem, SAD cause personal, health, societal, and occupational issues [5] which can directly influence and be influenced by the quality of life. The level of stress cited in self-reported examinations and surveys is inversely correlated with life quality and well-being [6].

Untreated poor mental health can cause distress among students and, hence, negatively influence their quality of lives and academic performance, including, but not limited to, lower academic integrity, alcohol and substance abuse as well as a reduced empathetic behaviour, relationship instability, lack of self-confidence, and suicidal thoughts [7]–[9].

A 21-item self-evaluating questionnaire, Beck Depression Inventory (BDI), is the most common tool used for diagnoses of depression [10]. A BDI-based survey in five developed countries in Europe (European Outcome of Depression International Network-ODIN in the United Kingdom, Netherlands, Greece, Norway, and Spain) concluded that overall 8.6% (95% CI, 7.95–10.37) of the resident population are dealing with depression [11]. Similar studies confirmed that about 8% of the population in developed and developing countries suffer from depression [12]. Data from systematic review studies revealed that this depression rate is much higher among university students and around one third of all students in the majority of the developed countries have some degree of SAD disorders; and depression prevalence has been increasing in academic environments over the past few decades [3].

Despite all the efforts to increase awareness and tackle mental health problems among university students, there is still an increasing number of depression and suicide among students [13], indicating a lack of effectiveness of the measures adopted. In addition to an increase in the prevalence of mental health issues, comparing students and non-college-attending peers demonstrated that the severity of psychological disorders that students receive treatment for has also increased [14]. For example, the rate of suicide among adolescents has increased significantly over the past few decades [15]. In fact, suicide as a result of untreated mental health is the second cause of death among American college students [16], emphasizing the importance of identifying and treating risk factors associated with SAD.

SAD can be manifested in different forms; however, some common overt symptoms include loss of appetite, sleep disturbance, lack of concentration, apathy (lack of enthusiasm and concern), and poor hygiene. Studying SAD is particularly important among university students who are future representatives and leaders of a country. Furthermore, most undergraduate students enter university at an early age; and dealing with SAD early in life can have long-term negative consequences on the mental and social life of students [3]. For example, a longitudinal study in New Zealand over 25 years demonstrated that depression among people aged 16–21 could increase their unemployment and welfare-dependence in long-term [17].

A better understanding of SAD among students in developed and developing countries not only helps governments, universities, families, and healthcare agencies to identify risk factors associated with mental health problems in order to minimise such risk factors, but also provides them with an opportunity to study how these factors have been changing in the academia.

This review aims to provide an updated understanding of risk factors associated with SAD among post-secondary undergraduate and college students in developed and developing countries by using existing literature resources available to answer the following question:

“Aetiology of depression and anxiety: What are risk factors associated with stress, anxiety and depression among university and college undergraduate students studying in developed and developing countries?”

It is worth mentioning that this review focuses on SAD risk factors of university students in developed and developed countries, and does not cover underdeveloped countries which can have their own niche problems (such as poverty). However, this review takes into account international students who migrate from underdeveloped countries to developed and developing countries to pursue their education.

## 2. Methods

### 2.1. Aims and objectives

The aims of this review were to identifying principal themes associated with depression and anxiety risk factors among university undergraduate students. The objectives of this review are to design a rigorous searching methodology approach by using appropriate inclusion and exclusion criteria, to conduct literature searches of publicly available databases using the designed methodology approach, to investigated collected literature resources to identify risk factors associated with the depression and anxiety which have not changed, and to identify principal themes associated with SAD risk factors among university undergraduate students.

### 2.2. Designed approach for literature review

A narrative review based on a comprehensive and replicable search strategy is used in this review. This approach is justified and preferred, over other approaches such as primary data gathering, because of the timescale of the research (2000 to 2020-temporal reasons), and extent of the research (developed and developing countries-spatial reasons).

### 2.3. Criteria for inclusion and exclusion of articles

Inclusion and exclusion criteria for articles and academic writings used in this review are as follows:

#### 2.3.1. Date

2000 to 2020 are included Academic writings which are published between in this review. Initially, during a pilot search, search strategies covered 1990 to 2020. However, the majority of the search results (more than 80% of the search results and more than 88% of applicable search results) were from 2000 to 2020, which indicates the importance of mental health issue and increased awareness over the past two decades. Therefore, for the final search, papers from 2000 to 2020 were included.

#### 2.3.2. Study design

Literatures included in this narrative review were primary research articles, review articles, systematic reviews, mini-reviews, opinion pieces, correspondence, clinical trials, and cases reports published in peer reviewed journals.

#### 2.3.3. Country

The narrative review was limited to developed and developing countries definition by the United Nations Department of Economic and Social Affairs [18]. Abstract and method sections of search results were screened to check the country of research.

#### 2.3.4. Language

Peer-reviewed articles published in English were only included in this narrative review.

#### 2.3.5. The explanation for papers exclusion

The main reason for papers excluded from consideration after search results was that they focused on intervention and therapies associated with SAD. Other reasons for exclusion was that studies were conducted on a mixture of undergraduate and graduate students or focused solely only graduate students. Studies which focused on other types of mental disorders such as eating disorders but did not focus on SAD were excluded too. The conducted search did not exclude any gender or specific age category.

### 2.4. Strategies used for search and limitations

In this review, a robust and replicable search strategy was designed to identify appropriate articles by searching PubMed, MEDLINE via Ovid, and JSTOR electronic databases. These databases were selected because they encompass biopsychosocial papers published on SAD. The date chosen for this search was for articles published between 2000 to 2020 which covers the past two decades. Once key articles were identified, a search for citation of those papers was conducted, and the bibliography of those papers were further screened to identify potential articles which can be relevant.

### 2.5. Search terminologies used

To conduct searches in databases mentioned above, the following search terms were used: students stress, anxiety, depression risk factors, university stress, anxiety, depression risk factors, student mental health developed and developing countries, students stress, anxiety and depression developed and developing countries. The operation AND was used to connect stress, anxiety, depression, mental health, developed, developing, countries, students. The search for each term was conducted in all fields (title, abstract, full text, etc.).

### 2.6. Screening, selecting search results, and data extraction

**Figure 1.**
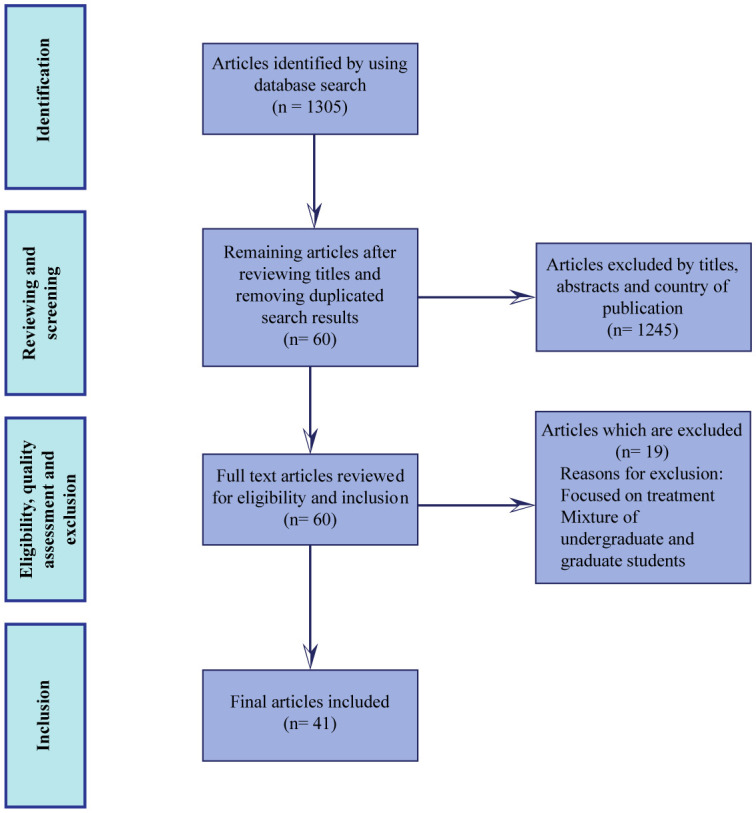
A flowchart demonstrating the procedure for identification, screening, exclusion and inclusion of the articles in this review.

The search results were exported into separate Excel and EndNote X8 files. Titles and abstracts from all articles were screened to determine their relevance to the topic of this review. Potentially relevant articles were fully read to establish their relevance. Each paper which was included according to the inclusion criteria described above was read fully. A word file was created to identify themes associated with SAD risk factors which is included in the Results. An initial search resulted in 1305 articles. The title and abstract of individual papers were read for relevance, resulting in 60 papers which were relevant for the research question asked in this review. All 60 papers were read completely, and from those, 19 were excluded based on the criteria mentioned before. Therefore, the total number of papers for consideration was 41. A flowchart explaining the procedure for identification, screening, eligibility, and inclusion of papers is shown in Figure 1.

**Figure 2.**
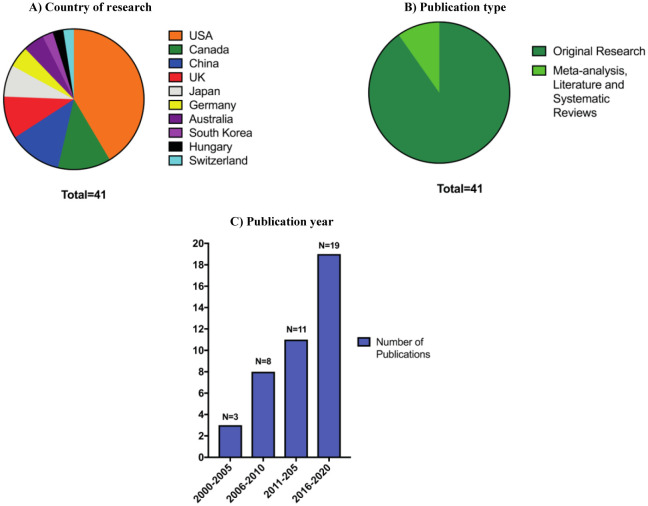
A quantitative summary of distribution of included papers. Analysis of search results were conducted to demonstrate (A) country of publication, (B) publication type, and (C) publication year.

Figure 2 provides a quantitative summary of the papers included in this narrative review. In terms of the distribution of the countries where the research was conducted, included papers were mainly articles which carried out studies in the USA (n = 17), followed by China and Canada (each n = 5), UK (n = 4), Japan (n = 3), Germany and Australia (each n = 2), South Korea, Hungary, Switzerland (each n =1) (Figure 2A). As for article types included in this review, original research articles, including quantitative and qualitative studies, which relied on obtaining data including cross-sectional studies, interviews, case-control studies, surveys, and questionnaire, were the highest (n = 37) followed by meta-analysis, literature and systematic reviews (Figure 2B). Another interesting observation was that although the search was carried out from 2000–2020, most papers were concentrated in the period from 2016 to 2020 (Figure 2C). This can be due to the reason that mental health is becoming more important over the past few years. Alternatively, a higher number of papers included from 2016 onward can be due to unintended selection bias. The smallest study covered in this narrative review was conducted on 19 students and the largest one on 153,635 students, adding up to 236,104 students, who were included in articles covered in this narrative review in total. Most studies on mental health, anxiety, and depression use standardised approaches such as patient-filled general health questionnaires, Pearling coping questionnaire, internally regulated surveys, BDI, DSM-IV symptomology, and general anxiety and burnout scales such as Maslach Burnout Inventory.

## 3. Results

### 3.1. Literature search results

**Figure 3.**
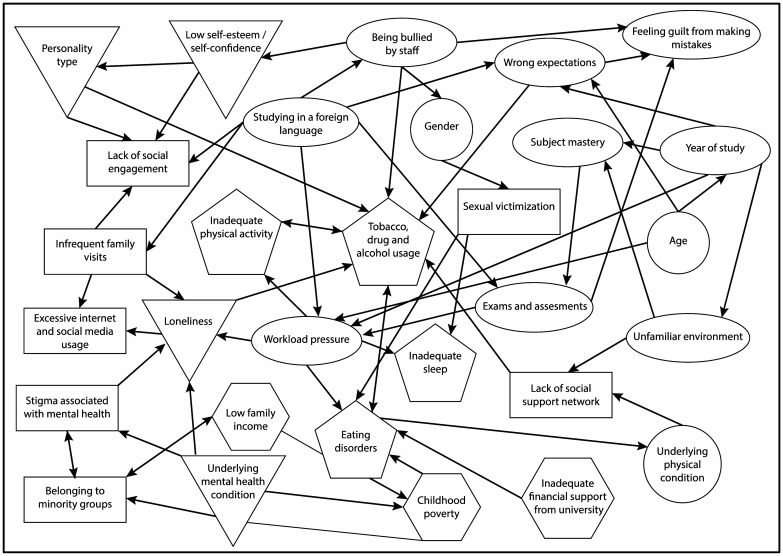
A concept map demonstrating a complex relationship between risk factors associated with SAD in university undergraduate students studying in developed and developing countries. Different themes are represented by different shapes: circle, biological; oval, academic; triangle, psychological, rectangle, social; pentagon, lifestyle; hexagon, financial. The size of shapes is arbitrary. Arrowheads indicate associations.

Following the search protocol shown in Figure 3, a list of included papers identified which can be found in the Table 1.

**Table 1.** A list of included articles with detailed information on authors, title, publication year, country, study type, sample size and type, and primary results.

Authors (Year)	Article title	Year	Country	Article type	Sample size	Sample type	Primary results
Armstrong and Young (2015) [63]	Mind the gap: Person-centred delivery of mental health information to post-secondary students	2015	Canada	Original research (survey)	271	Randomly selected university students	Social stigma and gender (being a male) is associated with a poorer mental health among university students.
Bovier et al. (2004) [31]	Perceived stress, internal resources, and social support as determinants of mental health among young adults	2004	Switzerland	Original research (survey)	1,257	Randomly selected university students	A lack of social support, unfamiliarity with the subject studying, underlying mental health condition and physical problems before entering the university are associated with increased level of stress.
Bradley [60]	Responding effectively to the mental health needs of international students	2000	UK	Original research (survey)	429	International students in the UK universities	Insufficient financial support, social isolation and academic pressure causes stress and anxiety among international students studying in the UK university.
Brockelman (2000) [40]	The interrelationship of self-determination, mental illness, and grades among university students	2009	USA	Original research (survey)	375	Undergraduate students at American universities	Ethnic group, low grades, age and wrong expectations from university diminishes students' determination, causing them to develop stress and anxiety.
Cai et al. (2015) [42]	Social Smoking and Mental Health Among Chinese Male College Students	2017	China	Original research (questionnaire)	1,327	Male university students in China	While tobacco smoking can increase social bonding among male Chinese students, it adversely affects mental health.
Wanda and Carla (2013) [27]	Stress, depression, and anxiety among undergraduate nursing students	2013	Canada	Original research (cross-sectional study)	882	Nursing students in Canadian universities	Academic pressure, fear of obtaining poor grades, lack of social support, problems with university staff and teachers, wrong expectation from the university, unfamiliarity with the new environment are risk factors of SAD.
Erschens et al. (2018) [64]	Professional burnout among medical students: Systematic literature review and meta-analysis	2018	Germany	Systematic literature review and Meta-analysis	N/A	N/A	High academic workload and career concerns are predictor for development of SAD.
Fortney et al. (2016) [25]	Prevalence of probable mental disorders and help-seeking behaviors among veteran and non-veteran community college students	2016	USA	Original research (survey)	765	Veteran and non-veteran students in American community colleges	Underlying mental and physical conditions before entering the university are risk factors for mental disorders. Social stigma significantly increases the risk.
Ghodasara et al. (2011) [28]	Assessing Student Mental Health at the Vanderbilt University School of Medicine	2011	USA	Original research (survey)	330	Second- and third-year medical students of Vanderbilt University	Having a family history of mental health increases the probability of depression during university years. Regular exercise can act as a protective factor against depression and anxiety.
Goodwill and Zhou (2020) [16]	Association between perceived public stigma and suicidal behaviors among college students of color in the U.S.	2020	USA	Original research (cross-sectional study)	153,635	American college students	Being a member of ethnic and religious minority group and experiencing social stigma are risk factor for depression and suicide ideation.
Hafen et al. (2006) [65]	Predictors of depression and anxiety in first-year veterinary students: a preliminary report	2006	USA	Original research (cross-sectional study)	93	First year Veterinary students	Homesickness, underlying physical condition and a lack of clear understanding of university expectations are among risk factors for anxiety and depression in Veterinary students in USA.
Hefner and Eisenberg (2009) [50]	Social Support and Mental Health Among College Students	2009	USA	Original research (survey)	1,378	Public university students	Being an international student, belonging to an ethnic or religious minority group and a lower socioeconomic status cause social isolation followed by SAD formation.
Hunt et al. (2010) [66]	Mental health problems and help-seeking behavior among college students	2010	USA	Literature review	N/A	N/A	Financial constraints, lack of social interaction, and concerns about adjusting to university life are associated with increased stress and anxiety among university students.
Ishii et al. (2018) [33]	What kinds of factors affect the academic outcomes of university students with mental disorders? A retrospective study based on medical records	2018	Japan	Original research (retrospective survey)	573	Undergraduate students at Tsukuba University in Japan	Infrequent family visits, lack of social engagement, gender (being a female), fear of poor grades and underlying mental health conditions are risk factors of students SAD among students in a Japanese university.
Jenkins et al. (2019) [46]	Exploring the implications of a self-care assignment to foster undergraduate nursing student mental health: Findings from a survey research study	2019	Canada	Original research (cross-sectional study)	89	Mixed university students in Canada	A lack of self-care and having an unhealthy lifestyle are risk factors for stress and anxiety among students. A lack of self-care can cause sense of guilt which can pose as another risk factor.
Kawase et al. (2008) [24]	Variables associated with the need for support in mental health check-up of new undergraduate students	2008	Japan	Original research (questionnaire)	8,287	Freshman students at Tokyo university	Personality type (high neuroticism and low extraversion), social isolation, fear of obtaining poor grades, and insufficient mastery of the subject are among strong predictors of mental health problems in Japanese universities.
Kitzrow (2003) [67]	The Mental Health Needs of Today's College Students: Challenges and Recommendations	2003	USA	Literature review	N/A	N/A	Increased workload and a lack of adequate counselling service cause mental health difficulties among students.
Lee et al. (2012) [32]	Mental Health and Coping Strategies among Medical Students	2012	South Korea	Original research (self-reported survey)	384	Korean medical students	Academic workload, concerns about academic career, and studying in a non-native language (English) are major risk factors associated with stress and depression. Students in different years of studies have different mechanisms for demonstrating their depression and lack of self-esteem.
Li et al. (2019) [57]	Sensitive Periods of Moving on Mental Health and Academic Performance Among University Students	2019	China	Original research (self-reported survey and questionnaire)	3,753	First year undergraduate students	Moving residence during childhood and loss of social network are associated with SAD problems in university life, especially among families with a lower socioeconomic status.
Macaskill (2012) [30]	The mental health of university students in the United Kingdom	2012	UK	Original research (cross-sectional study	1,197	UK undergraduate students	While subject of studying is not associated with mental health states of students, the year of studying correlates with the level of stress and anxiety.
Maser et al. (2019) [21]	Medical Student Psychological Distress and Mental Illness Relative to the General Population: A Canadian Cross-Sectional Survey	2019	Canada	Original research (cross-sectional survey)	4,613	17 Canadian medical schools	Subject of study (medicine) and gender (being a female) are associated with an elevated of stress and suicide ideation releative to general population.
McDougall et al. (2019) [54]	The Relationship Between Non-Consensual Sex and Risk of Depression in Female Undergraduates at Universities in Maritime Canada	2019	Canada	Original research (cross-sectional survey)	6,939	Female undergraduate students at Canadian universities	Sexual victimization of female undergraduate students significantly increases stress and anxiety, and if untreated, causes depression.
Meng et al. (2011) [51]	Susceptibility genes, social environmental risk factors and their interactions in internalizing disorders among mainland Chinese undergraduates	2011	China	Original research (genetic case-control study)	528	Healthy and genetically diseased undergraduate students	A lack of support from family and society and loneliness are associated with internalizing mental health disorders leading to SAD.
Michalec and Keyes (2013) [53]	A multidimensional perspective of the mental health of preclinical medical students	2013	USA	Original research (survey)	237	Pre-clinical medical students	Lack of social activity involvement is a risk factor for SAD. Students entering the university have different psychological needs that those graduating.
Miller-Graff et al. (2015) [26]	Typologies of childhood exposure to violence: associations with college student mental health	2015	USA	Original research (online questionnaire)	395	American universities	Exposure to violence during childhood and mental health associated with it poorer mental health of students in university.
Park et al. (2020) [61]	Understanding Students' Mental Well-Being Challenges on a University Campus: Interview Study	2020	USA	Original research (semi-structured interview)	19	Undergraduate students at a large American university	Social stigma associated with mental health discourages students from disclosing their mental health problems which can lead to SAD.
Ratanasiripong (2018) [22]	Mental Health and Well-Being of University Students in Okinawa	2018	Japan	Original research (cross-sectional survey)	441	3 Japanese universities	A lack of self-esteem, a lower socioeconomic background of the family and elevated academic workload are strong predictors of anxiety and stress among students.
Rosenthal et al. (2018) [47]	Alcohol consequences, not quantity, predict major depression onset among first-year female college students	2018	USA	Original research (prospective longitudinal survey)	412	First year female college students	Alcohol consumption is a major risk factor for university students, especially for females. Other factors include sexual victimization and lower socioeconomic status.
Scholz et al. (2016) [35]	Risk factors for mental disorders develop early in German students of dentistry	2016	Germany	Original research (survey)	163	Dentistry students in German universities	Academic workload and lack of engagement in social activities, especially during exam time, are risk factors of stress and anxiety.
Sprung and Rogers (2020) [68]	Work-life balance as a predictor of college student anxiety and depression	2020	USA	Original research (cross-sectional survey)	111	Private American universities	A lack of balance between academic and social life causes formation of anxiety and depression among students in American universities.
Stallman (2010) [34]	Psychological distress in university students: A comparison with general population data	2010	Australia	Original research (survey)	6,479	Undergraduate students from two large Australian universities	Financial difficulties, gender (being female), academic workload and year of study are predictors of stress.
Sznitman et al. (2011) [58]	The neglected role of adolescent emotional well-being in national educational achievement: bridging the gap between education and mental health policies	2011	USA	Meta-analysis	N/A	N/A	Financial difficulties, especially during childhood, makes students more vulnerable to having mental health problems in university.
Terebessy et al. (2016) [48]	Medical students' health behaviour and self-reported mental health status by their country of origin: a cross-sectional study	2016	Hungary	Original research (cross-sectional study)	1,683	Hungarian and non-Hungarian medical students studying in Hungary	A lack of sufficient physical activity is directly related to lower mental well-being among students. Increase in rigorous physical activity is associated with a better diet and a lower alcohol and tobacco consumption.
Thomas et al. (2020) [56]	Student Loneliness: The Role of Social Media Through Life Transitions	2020	UK	Original research (survey)	510	First year undergraduate UK students	Loneliness and subsequently excessive social media usage are associated with SAD.
Turner et al. (2007) [20]	Coventry university students' experience of mental health problems	2007	UK	Original research (questionnaire)	527	Coventry University undergraduate students	Being a member of ethnic minority increases mental health problem formation at university. Also, male students are less likely to seek help compared to females, which can exacerbate their mental health problems.
Usher and Curran (2019) [37]	Predicting Australia's university students' mental health status	2019	Australia	Original research (cross-sectional study)	2,326	Australian universities	Gender, age, negative health behaviours, lower level of physical activity and poor social emotional wellbeing are risk factors associated with SAD.
Vaughn et al. (2016) [59]	College student mental health and quality of workplace relationships	2016	USA	Original research (survey)	170	Part-time employed students	A lack of social support and problems with colleagues and peers in the working environment causes mental health problems among students.
Whitton et al. (2013) [52]	Committed dating relationships and mental health among college students	2012	USA	Original research (survey)	889	Undergraduate students	A lack of sufficient social interaction and support network such as being in a committed relationship can damage mental health directly and indirectly (by increasing alcohol consumption).
Yao et al. (2013) [55]	Freshman year mental health symptoms and level of adaptation as predictors of Internet addiction: a retrospective nested case-control study of male Chinese college students	2013	China	Original research (retrospective case-control study)	977	First year undergraduate students in North-western China University	Addiction to excessive and uncontrolled internet usage can cause stress, anxiety and depression among students. Prevalence of mental health problems was higher among first-year undergraduate students.
Zeng et al. (2019) [39]	Prevalence of mental health problems among medical students in China: A meta-analysis	2019	China	Original research (cross-sectional study)	30,817	Chinese medical students	While age and gender are not risk factors, studying a difficult subject such as medicine can be a predictor of mental health problem in students.
Zivin et al. (2009) [19]	Persistence of mental health problems and needs in a college student population	2009	USA	Original research (survey)	2843	Large American public universities	Social stigma associated with mental health acts as a risk factor for depression by prohibiting students from seeking help when under pressure.

### 3.2. Prevalence of mental health disorders in students

Literature showed that mental health problems are common phenomenon among students with a higher prevalence compared to the general public. For example, surveying more than 2800 students in five American large public universities demonstrated that more than half of them experienced anxiety and depression in their last year of studies [19]. Similarly, a survey of Coventry University undergraduate students in the UK showed that more than one-third of them had experienced mental health issues such as anxiety and depression over the past one year since they were surveyed [20]. In agreements with these results, Maser et al. [21] found that prevalence of mental health disorders including anxiety and depression was higher among medical students compared to the general non-student population of the same age. These studies demonstrated that the prevalence of SAD among students has remained higher than the average population over the past two decades.

SAD are not only prevalent among students, but also persistent. By conducting a follow-up survey study of students over two years, Zivin et al. [19] demonstrated that more than half of students retain their higher levels of anxiety and depression over time. This can be due to a lack of SAD treatment or persistence of existing risk factors over time.

### 3.3. Risk factors associated with stress, anxiety, and depression

SAD are multifactorial, complex psychological issues which can have underlying biopsychosocial reasons. Multiple risk factors which affect the formation of SAD among undergraduate university students in developed and developing countries were identified in this review. These factors can be categorized into multiple themes including psychological, academic, biological, lifestyle, social and financial. A summary of risk factors and their associated publications are shown in Table 2.

**Table 2.** A summary of risk factor groups associated with stress, anxiety and depression, and among undergraduate students in developed and developing countries, and publications relating to those risk factors. Six main themes of risk factors were identified: psychological, academic, biological, lifestyle, social, and financial.

Risk Factors	Related Publications
Psychological Factors
Low self-esteem and self-confidence	[22]
Underlying mental health condition before entering the university	[25],[26],[31],[33]
Personality type (high neuroticism and low extraversion)	[24]
Loneliness	[24]
Academic Factors
Fear of poor grades	[24],[27],[33],[34]
Workload pressure	[21],[24],[27],[28],[32],[35],[64],[66],[67],[68]
Exams and assessments	[35],[66]
Wrong expectations from the university and course	[27],[28],[40]
Negative relationship with teachers and staff	[27]
Studying in a non-native language	[32]
Lack of subject mastery	[24],[31]
Year of study	[22],[30],[32]
Guilt from making mistakes in workplace and assignments	[27]
Entering a new environment for workplace	[27]
Biological Factors
Underlying physical condition before entering university	[25],[26],[31],[33],[65]
Gender	[20],[21],[28],[33],[34],[37],[63]
Age	[37],[39],[40]
Lifestyle Factors
Tobacco smoking, alcohol consumption, and drug abuse	[24],[28],[42],[46],[47]
Lack of adequate physical activity	[27],[37],[48]
Eating disorders	[28]
Inadequate sleep	[28],[47]
Social Factors
Lack of a supportive social network	[27],[37],[50],[51],[52],[57],[59],[67]
Lack of social support from university	[31]
Infrequent family visits	[33],[65]
Lack of involvement in social activities	[33],[35],[37],[53],[57],[68]
Internet addiction and excessive social media usage	[55],[56]
Sexual victimization	[54]
Belonging to a race or religion minority group	[16],[20],[40],[50]
Social stigma associated with mental health	[16],[19],[25],[61],[63]
Financial Factors
Lack of adequate financial support	[22],[50],[57],[60],[66]
Low family income	[57]
Childhood poverty	[58]

#### 3.3.1. Psychological factors

Self-esteem, self-confidence, personality types, and loneliness can be associated with SAD among university students. Students who have a lower level of self-esteem are more susceptible to develop anxiety and depression [22]. Also, students with high neuroticism and low extraversion in five-factor personality inventory [23] are more likely to develop SAD during university years [24]. Other psychological factors such as feeling of loneliness plays important roles in increasing SAD risk factors [24]. Moving away from family and beginning an independent life can pose challenges for fresher students such as loneliness until they adjust to university life and expand their social network. Indeed, Kawase et al. [24] showed that students who live in other cities than their hometown for studying purposes are more likely to develop anxiety and depression.

Some students enter the university with underlying mental conditions, which can become exacerbated as they transition into the independent life at university. While depression is higher among university and college students compared to the general public, students with a history of mental health problems, such as post-traumatic stress disorder (PTSD), are more prone to development of anxiety and depression during their university lives compared to students who did not have such experience before starting their degrees [25]. Furthermore, exposure to violence in childhood either at the household or the community correlates with SAD formation later in life and at University [26]. Therefore, low self-esteem and self-confidence, having an underlying mental health condition before beginning the university, personality type (high neuroticism and low extravasation), and loneliness can increase the probability of SAD formation in students.

#### 3.3.2. Academic factors

Multiple university-related academic stressors can lead to SAD among students. One of these factors which was strongly present in many studies evaluated in this review was the subject of the degree. Medical, nursing, and health-related students have a higher prevalence of depression and anxiety compared to their non-medical peers [24],[27]–[28]. Medical and nursing students who have both theoretical duties and patient-related work usually have the highest level of workload among university students, consequently deal more with anxiety and depression [27],[29]. In addition, students who major in psychology and philosophy, similar to nursing and medical students, are more likely to develop depression during their studies compared to others [24]. These studies did not identify whether students who have underlying mental health conditions are more likely to choose certain subjects such as philosophy, psychology, or subjects which lead to caring roles such as nursing and medicine. Because of the nature of their work, medical and nursing students who deal with people's health can experience depression and anxiety as a result of fears of making mistakes which can result in harming patients [27]. Students with practical components in their degree are required to travel to unfamiliar places for fieldwork and work experience which can add to their stress and anxiety [27].

Also, some prospective students, especially those who study nursing and medicine, usually do not have a clear understanding of the curriculum and workload associated with the subject before entering the university, therefore, they can face a state of disillusionment once they begin their studies at university [27]–[29]. It is worth mentioning that not all studies found a significant correlation between the subject of study and SAD development [30]. This can be explained by differences in sample type and size which results in variations existing in the amount of workload and curriculum in similar subjects taught in various universities in different countries.

Studying a higher degree can be a challenging task which requires mental effort. Mastery of the subject can negatively correlate with self-esteem, anxiety, and depression among university students with students who have a mastery of subject demonstrating a lower level of stress and anxiety [31]. Also, students who study in a non-native language report the highest level of anxiety and depression during their freshman years, and their stress levels decrease during the subsequent study years [32]. This can be explained by the fact that students who are studying in a foreign language usually are those who have migrated abroad, therefore, require some time to adjust to their new lives. Different studies showed that the level of anxiety and depression among both international and home students could correlate with the year of study with fresher students who enter the university and students at the final year of their studies experience the greatest amount of anxiety and depression with different risk factors [22],[32]. While fresher students experience SAD because of challenges in adjustments to university life, past negative family experience, social isolation and not having many friends, final year students report uncertainty about their future, prospective employment, university debt repayment and adjusting to the life after university as major risk factors for their SAD [22],[32]. Therefore, a shift in SAD risk factors themes are observed as students make a progress in their degrees.

Students spend a significant portion of their time at university being engaged with their academic activities, and unpleasant academic outcomes can influence their mental health. Receiving lower grades during the time of studies can negatively influence students' mental health, causing them to develop SAD [33],[34]. Academic performance during undergraduate studies can determine the degree classification, which can, subsequently, influence students' opportunities such as employment success rate or access to postgraduate courses [27]. Conversely, both the number of students with mental health problem symptoms and the severity of students' SAD increase during exam time [35], reflecting a direct relationship between academic pressure and students' mental health states. However, the causal relationship is not well-established; it is possible that depression and associated problems such as temporary memory loss and lack of concentration [36] are reasons for poor academic grades or inversely, students feel stressed leading to depression because of their poor performance in their exams. A mutual relationship can exist between grades and mental health, as having a poor mental health can reciprocally cause students to get lower grades [34], leading to a vicious cycle of mental health and academic performance. Interestingly, students' sense of social belonging and coherence to the university community was reduced during exam periods [35]. This can be explained by the reduced participation rate of students in university social activities and clubs as well as an increased sense of competition with their peers. Furthermore, students interact directly and indirectly with teachers, lecturers, tutors, and other staff; therefore, the relationship between students and academic staff can influence students' mental health. A negative and abusive relationship with teachers and mentors can be another factor causing SAD among undergraduate students [27].

On the other hand, being a part-time student is a protective factor for anxiety and depression, and part-time students have better mental health compared to students with full-time status [34]. This can be explained by financial securities which have a source of income can bring or because part-time students are usually older than full-time students [34], and therefore, more emotionally stable. In conclusion, risk factors increasing SAD among university students include high workload pressure, fear of poor performance in exams and assessments, wrong expectations from the course and university, insufficient mastery in the subject, year of study, and a negative relationship with academic staff.

#### 3.3.3. Biological factors

Mental health can be influenced by ones' physical health. Presence of an underlying health condition or a chronic disease before entering the university can be a predictor of having SAD during university years [31],[33]. Students with physical and mental disabilities can be in a more disadvantaged position and do not fully participate in university life leading to SAD formation [33].

An association between gender and depressive disorders have been observed in several studies [21],[27],[34],[37]. Female students had a higher prevalence of SAD compared to male students. Interestingly, while female students demonstrated a higher level of SAD, the dropout rate of female students with a mental health problem from university was lower compared to their male counterparts [33]. On the other hand, while females are at a higher risk of developing depressive disorders, males with depressive disorders are less willing to seek professional help and ask for support due to the stigma attached to mental health [38], causing exacerbation of their problem over time [20].

Age can be another factor related to SAD. Younger students report a higher level of SAD compared to older students [34],[37]. However, other meta-analysis studies did not find a significant correlation between students' age and their mental health which can be due to sampling differences [39]. Some studies showed that while older undergraduate students have a higher determination to do well in the university [40], those who have family commitments are more prone to develop SAD during their degrees [27]. These discrepancies in findings can be explained by different sample sizes and types of studies which can be influenced by various confounding factors such as nationality, country of study, degree of studies, gender, and socioeconomic status. Similarly, a lack of correlation between depression prevalence and year of study is observed as some studies have reported a higher prevalence among earlier years of studies, while others have shown a higher prevalence among students as they move closer to the end of their studies [41]. These differences can be explained by different causes of depression in a different age; for example, while depression in younger adults can be due to changes in their environment and difficulties in adapting to a new life, older adults can have depression symptoms because of a lack of certainty for their future and employment. Nevertheless, differences exist between SAD risk factors associated with young and older students. Overall, biological risk factors affecting SAD include age of students, gender, and underlying physical conditions before entering the university.

#### 3.3.4. Lifestyle factors

Moving away from families and beginning a new life requires flexibility and adaptation to adjust to a new lifestyle. As most undergraduate students leave their family environment and enter a new life with their peers, friends, and classmates, their behaviour and lifestyle change too. Multiple lifestyle factors such as alcohol consumption, tobacco smoking, dietary habits, exercise, and drug abuse can affect SAD. Alcohol consumption is high among students with SAD [28]; a causal relationship was not been established in this study though.

Tobacco smoking is another risk factor associated with SAD which is common among students, especially students who study in Eastern developed and developing countries such as China, Japan and South Korea [24],[42]. Most students, especially male students, smoke because of social bonding and the rate of social smoking is directly correlated with SAD [24],[42]. Social smokers are less willing to quit smoking, and more likely to persist in their habit, resulting in long term negative physical and psychological health consequences [42]. Illegal substance abuse can be another factor important in SAD among young people [43]. Academic-related stress and social environment in university dormitories and student accommodations can encourage students to use illegal drugs, smoke tobacco and consume alcohol excessively as a coping mechanism, resulting in SAD [44]. Interestingly, students who perceived they had support from the university were feeling less stressed and were less at the risk of substance abuse [45], indicating the important role of social support in preventing and alleviating depression symptoms. This is of particular importance as a new social habit and behaviour adapted early during life can last for a long time. Furthermore, students who do not have a healthy lifestyle can feel guilt, which can worsen their SAD condition [46]. Interestingly, Rosenthal et al. [47] showed that negative behaviours resulting from alcohol consumption such as missing the next day class, careless behaviour and self-harm, verbal argument or physical fight, being involved in unwanted sexual behaviour, and personal regret and shame could be the main reasons for depression associated with drinking alcohol, rather than the amount of alcohol consumed.

In contrast, a moderate to vigorous level of physical activity can be a protective factor against developing SAD during university life [37],[48]. Students who have a perception of having inadequate time during their studies do not spend enough time for exercise and can develop SAD symptoms [27].

Another lifestyle-related risk fact associated with SAD is sleep. Many young people do not receive sufficient sleep, and sleep deprivation is a serious risk factor for low mood and depression [28],[47]. Self-reported high level of stress and sleep deprivation is common among American students [31],[49]. Insufficient sleep can act as a vicious cycle- academic stress can cause sleep deprivation, and insufficient sleep can cause stress due to poor academic performance since both sleep quality and quantity is associated with academic performance [28]. Overall, poor sleeping habit is associated with a decreased learning ability, increase in anxiety and stress, leading to depression.

Different negative lifestyle behaviours such as tobacco smoking, excessive alcohol consumption, unhealthy diet, lack of adequate physical activity, and insufficient sleep can increase the risk of SAD formation among university students.

#### 3.3.5. Social factors

Having a supportive social network can influence students' social and emotional wellbeing, and subsequently lower their probability of having anxiety and depression in university [27],[37],[50]. The quality of relationship with family and friends is important in developing SAD. Having a well-established and supportive relationship with family members can be a protective factor against SAD development, which, in turn, can affect the sense of students' fulfilment from their university life [27]. The frequency of family visits during university years negatively correlates with SAD development [33]. Family visits can be more challenging for international students who live far away from their families, therefore adding to existing problems of international students who live and study abroad.

In contrast, having a negative relationship with family members, especially parents, can cause SAD formation among students in university [51]. Similarly, having a strict family who posed restrictions on behaviours and activities during childhood can be a predictor of developing SAD during university years [51].

Also, it is shown that being in a committed relationship has a beneficial protective factor against developing depressive symptoms in female, but not male, students [52]. Interestingly, both male and female students who were in committed relationships reported a lower alcohol consumption compared to their peers who were not in committed relationships [52].

Involvement in social events such as participating in sporting events and engaging in club activities can be a protective factor for mental health [32],[37]. Assessing preclinical medical students' social, mental, and psychological wellbeing showed that while first year students demonstrate a decrease in their mental wellbeing during the academic year, they have an increase in their social wellbeing and social integration [53]. This can be explained by the time period required for fresher students who enter the university to adjust to the social environment, make new friends, and integrate into the social life of the university.

Access to social support from university is another factor which is negatively correlated with developing anxiety and depression [31]. It is worth mentioning that different universities provide different degrees of social support for students which can reflect on different anxiety and depression observed among students of different universities.

Importantly, sexual victimization during university life can be a predictor of depression. By surveying female Canadian undergraduate students, McDougall et al. [54] found that students who were sexually victimized and had non-consensual sex were at a higher chance of developing depression following their experience, emphasizing the importance of safeguarding mechanism for students at university campuses.

While the internet and social media can be great tools for maintaining a social relationship with classmates, pre-university friends and family members, it can have negative mental health effects. Excessive usage of social media and the internet during freshman year can be a predictor of developing SAD during the following years [55]. Students who have a higher dependence on the social media report a higher feeling of loneliness, which can result in SAD [56]. Students with internet addiction and excessive usage of social media are usually in first year of their degrees [55],[56] which can reflect a lack of adjustment to university life and forming a social network. Also, students who use social media more often have a lower level of self-esteem and prefer to recreate their sense of self [56], indicating an intertwined relationship between biopsychosocial factors in developing SAD among students.

Demographic status, ethnic and sexual minority groups including international Asian students, black and bisexual students were at an elevated risk of depression and suicidal behaviour [16],[50]. The frequency of mental health is usually more common among ethnic minorities. For example, Turner et al. [20] showed that ethnic minority students report a higher level of anxiety and depression compared to their white peers; however, they do not ask for help as much. Other studies supported these findings by showing that students from ethnic and religious minorities, regardless of their country of origin and country in which they study, have a higher prevalence of anxiety and depression compared to their peers [50]. Also, students' expectations from university can be different among ethnic minorities students, and most of them do not have a sufficient understanding of the services that university can provide for them [40].

Therefore, lack of support from family and university, adverse relationships with family, lack of engagement in social activities, sexual victimization, excessive social media usage, belonging to ethnic and religious minority groups, and stigma associated with the mental health are among risk factors for SAD in university students.

#### 3.3.6. Economic factors

Students' family economic status can influence their mental health. A low family income and experiencing poverty can be predictors of SAD development during university years [22],[50],[57],[58]. A higher family income can even ameliorate negative psychological experiences during childhood, which can have long-term negative consequences on the mental health of students once they enter university [57]. Also, experiencing poverty during childhood can have negative long-term consequences on adults, leading to SAD development during university life [58].

Some students take up part-time job to partially fund their studies. Vaughn et al. [59] showed that relationship of employed students with their colleagues in the workplace could affect students' mental health; and those students who had a poor relationship with their colleagues had worse mental health. However, it is worth mentioning that a causal relationship was not established. It can be possible that students who have poor mental health cannot get along with their co-workers, resulting in an adverse working relationship.

Because of paying higher tuition fees and less access to scholarships and bursaries available, international students can have more financial problems, causing a higher degree of anxiety and depression compared to home students [60].

Lack of adequate financial support, low family income and poverty during childhood are risk factors of SAD in students of undergraduate courses in developed and developing countries.

### 3.4. Stigma associated with mental health

While efforts have been put to reduce the stigma associated with receiving help for mental health problems, this still remains a challenge. For example, more than half of students who had SAD did not receive any help or treatment for their condition because of the stigma associated with mental health [19],[61]. This is not related to the awareness of the availability of mental health resources which was ruled out by authors, as most of the students who did not receive any help for their mental health problem were aware of available help and support to them [19].

Furthermore, the social stigma associated with receiving help for mental health problems was significantly associated with suicidal behaviour, acting as a preventive barrier to seek help (planning and attempt) [16]. Among students, those with a history of mental health problem such as veterans with PTSD are less likely to seek for help compared to non-veteran students [25], making them more susceptible to struggling with untreated mental health.

## 4. Discussion

This review tried to identify and summarise risk factors associated with SAD in undergraduate students studying in developed and developing countries. The prevalence of SAD is high among undergraduate university students who study in developed and developing countries. Untreated SAD can lead to eating disorders, self-harm, suicide, social problems [28]. Similar to a complex society, differences exist among students leading to complicated risk factors causing SAD. Because different themes influencing SAD has been investigated as a distinct body of research by different literature, a concept map is created to demonstrate the relationship between various risk factors contributing to the development of SAD in undergraduate students in developed and developing countries. Figure 3 bridges risk factors concepts between different literature. For most students, entering university is a new step in their lives which is associated with certain challenges such as moving into independent accommodation, social identity, financial management, making decisions, and forming a social network. Different students have different needs depending on the stage of their degree, which needs to be fulfilled. For example, coping with a new university life style can be a challenging task for students who enter the university. This becomes more significant for students moving abroad for their studying who need to adapt to a new lifestyle, speak in a different language, and live away from their families. In agreement with this, different levels of anxiety and depression with different risk factors are observed among students as they progress in their degrees. On the other hand, students who are finishing their degrees can have SAD because of uncertainties about their future.

Students learn different modules in different degrees and have different abilities. Mastery of the subject can be a factor affecting students' sense of self-esteem, influencing their anxiety level and developing depressive symptoms. This partially can explain changes in risk factors observed as students' progress in their degrees. Final year students who adjust to the university environment and develop mastery in their subject can deal with academic pressure better compared to freshers who transform from secondary school life to university lifestyle.

Students can come with a varied and challenging background such as those who experienced household and domestic violence, sexual abuse, and child poverty which can make them susceptible to developing anxiety and depression once academic pressure is mounted. As universities are diverse environment which enrol students from different socioeconomic background and different cultures, universities need to identify risk factors for different students and have robust plans to tackle them to provide a fostering environment for future leaders of the society. Therefore, early mental health screening can help to identify those students who are at risk to provide them with special and additional mental health support. Students not only should be screened for their mental health state as they enter university, but also regular follow up check-ups should be conducted to monitor their conditions as they progress in their degrees to detect early signs of SAD.

University and academic staff can play a significant role in either exaggerating or ameliorating risk factors associated with anxiety and depression. While teachers and mentors can support students to cope with SAD, they can be a source of problem too by discriminating, bullying, and hampering students' progress.

Managing finance and expenses can be a challenging task for students who are stepping into an independent lifestyle and need to pay for their tuitions in addition to their maintenance fees. While some students have access to private funding, bursaries, and scholarships, other students receive loans which they need to pay back or have part-time jobs to meet their expenses. Students who work need to have a work-life balance and the time spent in their jobs can affect the quality of their education.

Fresher students try to establish their social network and might feel isolated, which can push them to excessive usage of social media to fill their social gap. While internet addiction and excessive usage of social media can have a negative impact on students' mental health, technology, such as mobile phone applications can be used in universities campuses to promote a healthier lifestyle and reducing risk factors among students. For example, many students refuse to receive face-to-face mental health counselling support during their anxiety and depression due to stigma associated with disclosure of mental health issues. Providing students with anonymized counselling services through mobile phone applications can be one way of delivering help to students at universities.

With the advent of social media platforms such as Facebook, Twitter, Instagram, TikTok, etc., more and more students rely on such networks for socialisation. While the internet and online platforms can have beneficial consequences for students, such as rapid access to a variety of online learning resources and keeping in contact with friends and families, excessive usage of social media and internet can have negative consequences on students' academic performance. A poor mental health state at the beginning of university life is a predictor of internet addiction later during the degree. Heavy reliance on the internet can be a coping mechanism for students with anxiety and depression to overcome their mental health problems.

As governments and educational bodies in developed and developing countries are emphasising recruitment of ethnic minority students to university to increase the range of equality and diversity among students, it is important to consider the mental health of those students in the university as well. Students in minority groups such as black, international Chinese and bisexual student report a higher level of anxiety and depression compared to other non-minority group students. This can be due to either pre-existing conditions which student experience before entering the university, and can be exacerbated during the university, or can be because of problems which can develop during university life.

Also, more mental health support is available in universities as the number of university students is increasing, and there is a better understanding of the importance of mental health in academia; however, the stigma associated with mental health has not changed proportionately.

While research and understanding of mental health have changed significantly over the past two decades and many more articles are present, risk factors associated with SAD remain unchanged.

One caveat with studies of mental health among student is that most studies have been conducted among medical and nursing students and neglected non-medical students. One potential explanation for the tendency to conduct depression surveys among medical students is the higher response rate as medical students are more willing to fill out the questionnaires and surveys. It is understandable that students studying medical subjects, who directly interact with the public and treating them once they enter the healthcare profession should have a reasonably sound mental health to be able to conduct their duties, but it does not justify neglecting the mental health of other students. Therefore, more research on mental health and risk factors associated with SAD of non-medical students is required in the future.

Another caveat with most mental health studies is that they are based on self-reports and surveys. Different people can have different perception and understanding of mental health and anxiety, and many confounding factors can influence the response of participants in the time of participation. Furthermore, students with severe mental health conditions are less likely to participate in any activity including surveys and questionnaires, leading to a non-response bias.

Another area which requires improvement in future studies of mental health is the categorisation of different types of depression and their severity. Depression and anxiety are a spectrum which can comprise of minor and major symptoms; however, most studies did not specify the scale of depression in their findings. Furthermore, while various risk factors were identified, a causal relationship between mental health and behaviours were not established.

While counselling services provided by universities in Western countries such as the UK and USA have increased over the past few years [62], it is still not clear how effective such services are; therefore, more research is required to assess the effectiveness of counselling services at universities.

Therefore, a better understanding of the aetiology, associated factors is required for an effective intervention to reduce the disease incidence and prevalence among students in the population and providing them with a fostering environment to achieve their potential.

University undergraduate students are at a higher risk of developing SAD in developed and developing countries. Promoting the mental health of students is an important issue which should be addressed in the education and healthcare systems of developed and developing countries. Since students entering university are from different socioeconomic background, screening should be carried out early as students.

A personalized approach is required to assess mental health of different students. In addition, a majority of mental health risk factors can be related to the academic environment. A personalised, student-centred approach to include needs and requirements of different students from different background can help students to foster their talent to reach their full potential. Furthermore, more training should be provided for teaching and university staff to help students identify risk factors, and provide appropriate treatment.

## 5. Conclusion

Despite all the efforts over the past two decades to destigmatise mental health, the stigma associated with mental health is still a significant barrier for students, especially male students and students from ethnic and religious minorities to seek help for SAD treatment. Universities need to continue to destigmatise mental health in university campuses to enable students to receive more in campus support by providing designated time for positive metal health activities such as group exercise, physical activities, and counselling services. There is no shortage of athletic and group activities in form of clubs and social classes in most universities in developed and developing countries; however, more incentives such as athletic bursaries and prizes should be provided to students to encourage their participation in such activities which can act as protective factors against SAD development. Therefore, universities need to allocate more resources for sporting and social activities which can impact the mental health of students. Furthermore, an increase in mental health problems in universities has created a huge burden on university counselling services to meet the demands of students. More novel approaches, such as online counselling services can help universities to meet those increased demands.

Students in different years of studies deal with different risk factors from the time that they enter the university until they graduate, therefore, different coping strategies are required for students at different levels. Universities should be aware of these risk factors and implement measures to minimise those factors while providing mental health treatments to students.

Future studies are required to investigate long-term effects of experiencing SAD on students. A longitudinal study with a large randomly recruited sample size (different age, sex, degree of study, – socioeconomic status, etc.) is required to address how students' mental health change from entering the university until they graduate. Also, more extended follow up studies can be included to address the effect of depression and poor mental health on people's lives after they graduate from the university.
